# Stop hate crimes and racism

**DOI:** 10.1186/s13045-021-01078-2

**Published:** 2021-04-16

**Authors:** Chinese American Hematologist and Oncologist Network (CAHON)

**Affiliations:** CAHON, 555 East Wells Street, Suite 1100, Milwaukee, WI 53202 USA

The Chinese American Hematologist and Oncologist Network (CAHON), whose mission is to foster communications among Chinese American medical professionals for the delivery of high-quality health care to patients with cancer and blood diseases, vehemently condemns all forms of hatred, violence and racism in our society. While, sadly, these are not new issues, the recent uptick in discrimination and hate crimes against the Asian American and Pacific Islander community is devastating and unsettling to us all as a society.

The increasing hate crimes and violence towards Asian Americans and Pacific Islanders have incited fear and terror within our community. All human beings are free and equal in dignity and rights. No one should have to fear for their safety or be victims of any kind of physical or emotional attacks due to their physical appearance or cultural and ethnic background. The right to freedom from discrimination is a fundamental human right—one that we must uphold.

Built upon and thriving on immigration, the USA has always been a beacon of hope and justice to attract talented and hardworking immigrants from all over the world. Immigrants and their descendants have been and will continue to make tremendous and incontrovertible contributions to the growth and prosperity of the USA. Over 30% of the Nobel Prize Laureates in the USA during 1901–2013 were born in other countries, including 6 from China and 5 from Japan [1]. Asian Americans and Pacific Islanders are a vital and integral part of this diverse nation.

We call on you to denounce in the strongest terms all forms of discrimination and crimes against any racial/ethnic group, including Asian Americans and Pacific Islanders. We call on you to pursue racial equality and tolerance in the USA and beyond. We call on you to stand strong and together with us.
